# Development of the educational leadership scale for nursing students: a methodological study

**DOI:** 10.1186/s12912-023-01254-4

**Published:** 2023-04-10

**Authors:** Funda Karaman, Derya KavgaoĞlu, Gülay YILDIRIM, Mahruk RASHIDI, Gülşah ÜNSAL JAFAROV, Hina ZAHOOR, Neşe KISKAÇ

**Affiliations:** 1 Faculty of Health Sciences, Department of (English) Nursing, İstanbul Gelişim University, İstanbul, Turkey; 2 Faculty of Health Sciences, Department of Social Work İstanbul, İstanbul Gelişim University, İstanbul, Turkey; 3 Department of Nursing, Trakya University, Kesan Hakki Yoruk School of Health, Edirne, Turkey; 4 Faculty of Health Sciences, Department of Nursing, İstanbul Gelişim University, İstanbul, Turkey; 5 Faculty of Health Sciences, Department of (English) Social Work, İstanbul Gelişim University, İstanbul, Turkey

**Keywords:** Nursing student, Educational leadership scale, Healthcare, Validity, Reliability

## Abstract

**Background:**

Educational leadership is one of the most demanding skills for healthcare staff to enhance the quality of health care. There is a need for a scale to evaluate the educational leadership levels of nurses. The objective of this study was to develop and examine the validity and reliability of the Education Leadership Scale for Nursing Students.

**Methods:**

Data were collected from 280 Turkish nursing students. The validity and reliability of the tool were confirmed with exploratory and confirmatory factor analysis, Cronbach’s alpha and Pearson correlation. The scale was developed in five stages (reviewing the literature, developing items, sending scale items to the experts for content validity index, piloting test with students, performing the validity and reliability analysis of the tool).

**Results:**

The Educational Leadership Scale for Nursing Students consisted of 19 items and a three factor structure. Confirmatory factor analysis results showed that there was a sufficient model fit. Construct validity was verified, and Cronbach’s α level of all factors was found to be greater than 0.70.

**Conclusions:**

The currently developed scale can measure the educational leadership characteristics of nursing students.

## Introductıon

Effective leadership does not require a title. Leadership can occur in daily work, and occurs in cooperation with other professionals within the education and health service. For instance, leadership in the areas of education, research or clinical experience. To achieve more effective outcomes, leadership and management skills are now an expectation and a requirement for health care education [1]. Similarly, Educational leadership for nursing is an important dimension. Contemporary nursing demands that nurses have knowledge and skills in a variety of areas. The primary role definition of nursing, which focused mainly on providing care and comfort, has today left its place to a role definition that focuses on caregiver, decision-maker, protective and patient rights advocate, manager, rehabilitative, communicative, educational and empowering function [2].

Nurses are the educational leaders who lead the learning and teaching activities of their target groups, taking into account the structural, individual, cultural, political, and pedagogical dimensions of the health or educational institutions. For this reason, it is so important that nurses, who have roles and responsibilities within health and educational institutions, have educational leadership features [3]. Educational leadership skills can be learned and mastered over time. It’s very important to acquire those basic skills at school as soon as possible. Moreover, nursing practice consists of cognitive, social-interpersonal (affective) and psychomotor skills and all these competencies are necessary for implementing interventions.

Many researches believe in the assertion that nurse is responsible for knowing when to prefer which of these methods and having the necessary theoretical knowledge and practical skills to apply them [4–19]. When the literature on educational sciences is examined, it is possible to say that an effective educational leadership will occur with the effective guidance of the leader in scientific, educational and visionary terms [20–34]. For this reason, nurse educators have a great responsibility and while giving their students the most important theoretical knowledge and application opportunities for their profession, they should instill a desire and expectation for new learning in the coming years [2].

When the relevant literature was searched, a similar scale measuring educational leadership characteristics in nursing was not encountered in the perspective of Bloom’s taxonomy. Therefore, the present research focuses on the educational leadership dimension of nurses. The aim of the study is to develop “the Educational Leadership Scale for nursing Students and examine its validity and reliability”. It is believed that this scale will make an important contribution to the literature on educational leadership in nursing, and also provide self reporting tool in this context.

### Methods

#### Study design

This study was intended to be a methodology study.

### Study sample

The study was carried out between February and April 2022, in a private nursing school in Istanbul, which offered a bachelor’s degree level. The number of students was 420. Previous researches have indicated that the study population should be at least 5 to 20 times the number of the total item [35–37]. The scale consisted of 36 items, the required sample size was at least 180 participants. Therefore, the draft scale were distributed to 320 students (40 students were absent or did not agree to participate). The sample consisted of 280 students who agreed to participate in the research, filled the forms correctly and provided the appropriate data. The response rate was 73.6%.

### Data collection instrument

Data instrument has 2 sections.

Section A contains sociodemographic features.

- Age.
- Gender.

Section B is based on 36 items of the tool developed by the researchers.

### Study process of educational leadership scale for nursing students

The tool was developed in five stages. These stages are literature review, developing items, sending scale items to the experts for content validity index and then making revisions, piloting test with students and then applying main survey, performing the validity and reliability analysis of the scale [38].

#### Stage I

The relevant literature was reviewed in detail. For the present study the researchers reviewed different databases as the other researchers followed in their resesarches. “Educational Leadership Scale for Nursing Students” was prepared by making use of the educational sciences literature. The three-subscale model of the scale structure was created based on the gradual classification of learning objectives, which was first prepared by Bloom et al. in 1956 and revised by his colleagues in 2001 [2, 4–6, 8, 10, 13, 15, 16, 19].

There are various classifications on how to create and measure outcomes in education. In terms of facilitating and guiding the determination of the education outcomes, the taxonomies that were revealed in the 1950s-60s attracted attention all over the world and became an indispensable tool despite various criticisms. Taxonomy (1956), which is widely accepted in the gradual classification of educational goals, has been translated into 22 languages in the world since its publication [5].

In this classification, the objectives in a certain field are ordered from easy to difficult, from simple to complex. The simplest behaviors are at the bottom of the progressive list, while the most complex behaviors are at the top of the list. The gradual classification of targets is made in three areas. These areas are the Cognitive Area, the Affective Area and the Behavioral Area [16]. Bloom Taxonomy has been known and used in Turkey since 1972 [8].

According to Bloom, people are born with mental equipment related to learning and they have an unlimited learning capacity. However, their training process determines how much of their equipment and limits they can use. Therefore, when appropriate learning conditions are provided, each individual can learn almost anything that falls within their learning area. Individual differences in learning are not related to learning less or more, but stem from the individuality of individuals’ learning styles, interests, motivations and pace. In this context, the taxonomy of educational goals is a classification framework that expresses what students’ goals for learning or our expectations are as a result of teaching [15]. Taxonomy is a strategy that will facilitate and systematize learning in Nursing, as in all branches of science. For this reason, this systematic strategy was used on the theoretical ground in the development of a self-report tool that will help develop educational leadership competency in nursing.

#### Stage II

In the second stage, an item pool was created by the authors aligning the Bloom Taxonomy and Educational leadership. While creating the item pool, Bloom taxonomy domains (cognitive, emotional and behavioral) were used. Accordingly, while the “scientific and visionary” subscales of leadership included items from the “cognitive, affective and behavioral” domain outcomes, “affective and behavioral” domain outcomes were included in the “instructional leadership” subscale. The classification of the scale items according to Bloom’s Taxonomy is clearly presented in the table below (Table 1). In order to write the items to be included in each subscale called scientific, instructional and visionary leadership, the relevant literature was examined in detail and the following conclusions were reached.

**Table 1:** Theoretical Modeling for the Educational Leadership Scale for Nurse Students

Progressive Classification of Targets According to Bloom’s Taxonomy	Educational Leadership Development Area	Educational Leadership Subscales	Item number	Educational Leadership Characteristics
1	Emotional	Scientific Leadership	1.	I believe that I will be a professional role model for nursing profession candidates.
1	Emotional	Instructional Leadership	20.	I believe that the training contents should be designed in accordance to the needs of nursing profession.
1	Emotional	Visionary Leadership	35.	I think it is essential to develop cooperative skills in nurse candidates.
1	Emotional	Visionary Leadership	36.	I think classroom education should be conducted in a democratic climate.
2	Behavioral	Scientific Leadership	4.	I am willing to share my field related knowledge.
2	Behavioral	Instructional Leadership	13.	I believe that the field related learning modules should design in a way that develop interest for nurse candidates.
2	Behavioral	Instructional Leadership	17.	I believe that educational materials should be designed in accordance to learning objectives.
2	Behavioral	Instructional Leadership	18.	I believe that the most appropriate method should be chosen to determine the training needs of nurse candidates.
2	Behavioral	Instructional Leadership	19.	I believe that the learning progress of nurse candidates should be evaluated frequently. Item 20 (9)
2	Behavioral	Visionary Leadership	21.	I believe that the standards of National Core Program for Nursing should follow to plan educational program
3	Cognitive	Visionary Leadership	22.	I believe that standards of International nursing education program should follow to prepare the educational program.
3	Cognitive	Visionary Leadership	24.	I believe that nursing educational program should design in a way that facilitate learning. .
3	Cognitive	Visionary Leadership	26.	I believe that technology should be used in vocational training.
3	Cognitive	Visionary Leadership	27.	I believe that the most appropriate assessment method should be used in the evaluation of vocational training.
3	Cognitive	Visionary Leadership	30.	I think it is very important to adopt professional ethics in nursing training.
3	Cognitive	Visionary Leadership	32.	I believe that learning module should be designed in accordance to nursing professional ethics.
3	Cognitive	Visionary Leadership	33.	I think it is important to develop and improve the communication skills of nurse candidates.
3	Cognitive	Scientific Leadership	2.	I frequently update my knowledge regarding my profession.
3	Cognitive	Scientific Leadership	3.	I follow the scientific activities related to my field.

1. Cognitive skills are an important competency for the nurse. These skills include nursing knowledge and form the academic background of the nurse. For example, nurses should know the rationale for their therapeutic interventions, understand normal and abnormal psychology and psychological responses in this direction, identify the patient/client’s learning and discharge needs, and recognize the need for preventive nursing actions [2]. Professional attitude and competence on an academic basis, keeping professional knowledge up-to-date by actively following the intellectual activities related to the profession, can be classified as cognitive competencies that must be acquired with the “scientific leadership subscale”. Education with a professional vision for cognitive development, correct selection of materials and methods in education, gaining professional ethics awareness can be classified as cognitive competencies that must be acquired with the “visionary leadership subscale” [4–6, 8, 10, 13, 15, 16, 18, 19]. Affective or, in the nursing profession, social-interpersonal skills in a broader sense are essential for effective nursing action. The nurse’s communication with the patient, family, and other members of the healthcare team should be clear and unambiguous. Education and counseling of the patient/client should be based on the level of emotional response to the disease and treatment. The use of interpersonal skills enables the nurse to perceive the verbal and nonverbal communication of the patient/client [2]. In this meaning, affective competencies to be acquired in “scientific, educational and visionary leadership subscales” can be considered respectively as nurses being a right role model for career candidates, sensitivity to the real educational needs of learners, and creating an educational environment including cooperation and democracy [4–8, 13–19].
2. Psychomotor skills are directly related to care needs in nursing. Practices such as dressing, injection, tracheostomy can be given as examples. Nurses have professional responsibilities to accurately complete these skills. Some of these skills may be new to nurses. In this case, the nurse should seek help and supervision when necessary, ensure that the patient/client receives the treatment safely, and be able to accurately assess the current level of competence [2]. In this sense, it can be considered as the execution of the behavioral competencies that should be acquired in the “scientific, educational and visionary leadership subscales” with a vision in line with the quality standards of the education programs [4–16, 18, 19].

Among the items in the first list prepared, the ones that were determined to have a consensus on the important theorists of the field of educational sciences were selected and a new short list was created by eliminating the others. The final item pool was created by taking the opinions of subject matter experts from the disciplines of nursing and educational sciences in order to control the items in this short list with an interdisciplinary perspective. The factors of educational leadership are grouped under three main headings: scientific, instructional and visionary leadership. The “Educational Leadership Scale for Nursing Students” is designed to show the level of educational leadership according to the students’ responses to the scale, in proportion to the high scores they will receive in each factor and in the overall scale. Finally, the tool was organized with 36 items on a 5-point Likert scale (1:Strongly disagree, 2:Disagree, 3:Undecided, 4:Agree, 5:Strongly agree).

#### Stage III

The items of the scale were examined by three experts in the field of nursing, four experts in the field of educational sciences. Davis method was performed in the evaluation of the scale items by the experts [39], and the items in the scale were evaluated by the experts as (a) “Extremely appropriate”, (b) “Quite appropriate”, (c) “Slightly appropriate” and (d) “not applicable”. With this method, the number of people who chose the first two options was divided by the total number of experts. Content Validity Index (CVI) values of the tool were calculated and the values were found to be 0.80 and above. There was no amendment based on feedback and it was decided that the scale would consist of 36 items.

#### Stage IV

The pilot test provided obtaining feedback about the developed scale from students and also assessed the feasibility of the main survey. Before the main survey, in order to test the suitability and comprehensibility of the draft scale by the individuals, a pilot study was conducted with 30 students, and their opinions were received about determining the confusing or misleading items. No problems were experienced with students’ understanding of scale. After pilot study, the main study was conducted as there was no problem in students’ understanding of the scale.

#### Stage V

Exploratory Factor Analysis (EFA) was performed for determining the validity of the scale. In this analysis, the factor loading of the scale was determined as at least 0.30 [40]. After performing the EFA, for the accuracy of the structure was conducted confirmatory factor analysis (CFA). Internal consistency of the scale was evaluated by the Cronbach’s alpha and the stability reliability of the scale was also evaluated by the test-retest method.

### Statistical analysis

The data of the scale were analyzed by using IBM SPSS Statistics 21.0 and SPSS AMOS 27.0 program. The statistical results were considered significant at a 95% confident interval and p < 0.05. The analysis methods were indicated in phase V of the article.

### Ethical considerations

The study was ethically approved by Gelisim University Ethical Review Committee (no.10/2020). All procedures performed in studies involving human participants were in accordance with the ethical standards of the Declaration of Helsinki. Before starting the survey, all students informed about the athe objectives and methods of the study, the right to withdraw participation from the study, and use and confdentiality of the collected data. No scale was distributed to students who did not agree to participate. Informed consents were obtained before applying the tool.

## Results

### Demographic characteristics of students

In this study, the mean age of the students was 20.74 ± 1.88 (18–36), 66.8% were female and 35% were in second-year students.

### Results of the validity and reliability study

#### Construct validity analysis

EFA and CFA were performed to analyze the construct validity of the scale. Principal components analysis and varimax rotation approaches were used for factor analysis.

In the study, EFA was carried out for the 36 items. The KMO measure was 0.920, Bartlett’s sphericity was statistically significant (χ2 = 2652.138, df = 171, p < 0.001). Anti-image r values of the tool were found between 0.86 and 0.95. The first-factor analysis was conducted with 36 items. Items (5, 6, 7, 8, 9, 10, 11, 12, 14, 15, 16, 23, 25, 28, 29, 31, 34) with factor load values less than 0.30 were removed from the scale. Factor analysis was repeated with 19 items, it was found that the items were collected in 3 sub-scales and each item was included in only one sub-scale. These 3 subscales explained 58.64% of the total variance of the scale (Table 2).

**Table 2:** Total Variance Explained

Factors	Initial Eigenvalues					Extraction Sums of Squared Loadings				Rotation Sums of Totals of Squared Loadings		
	Total	Explained Variance %			Cumulative %	Total		Explained Variance %	Cumulative %	Total	Explained Variance %	Cumulative %
1	7.990		42.052	42.052		7.990	42.052		42.052	5.511	29.007	29.007
2	1.641		8.638	50.690		1.641	8.638		50.690	3.142	16.535	45.542
3	1.511		7.952	58.642		1.511	7.952		58.642	2.489	13.100	58.642
4	0.924		4.863	63.505								
5	0.759		3.997	67.502								
6	0.733		3.858	71.359								
7	0.646		3.400	74.759								
8	0.622		3.276	78.035								
9	0.551		2.900	80.935								
10	0.539		2.838	83.773								
11	0.519		2.733	86.506								
12	0.454		2.387	88.894								
13	0.403		2.120	91.014								
14	0.338		1.778	92.792								
15	0.316		1.664	94.456								
16	0.292		1.538	95.994								
17	0.290		1.525	97.519								
18	0.246		1.296	98.815								
19	0.225		1.185	100.000								

**Fig. 1:**
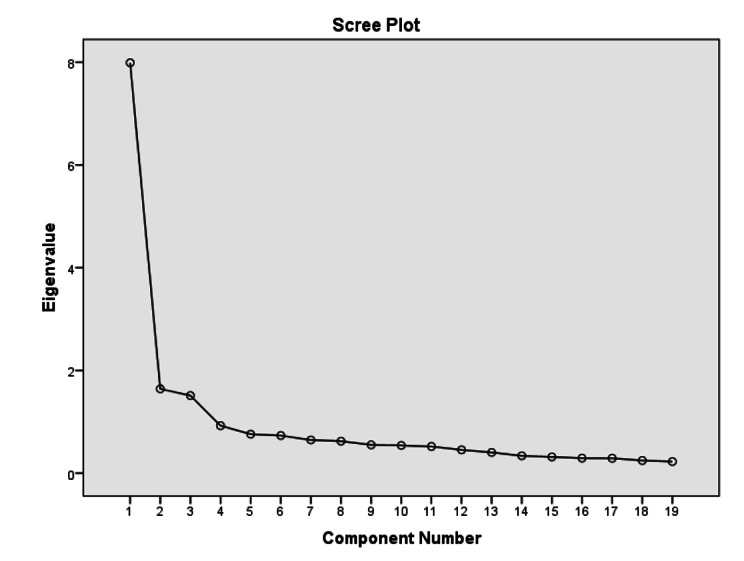
Scree Plot

In the scree plot graph, it was found that the break point of the curve coincided with the third factor and then the curve progressed at the same level (Fig. 1).

The rotated component matrix indicated that the scale had 3 factors with an eigenvalue higher than 1, and items of each factor had loading above the acceptable limit (the lowest item load value was 0.50; the highest item load value was 0.80).

**Fig. 2:**
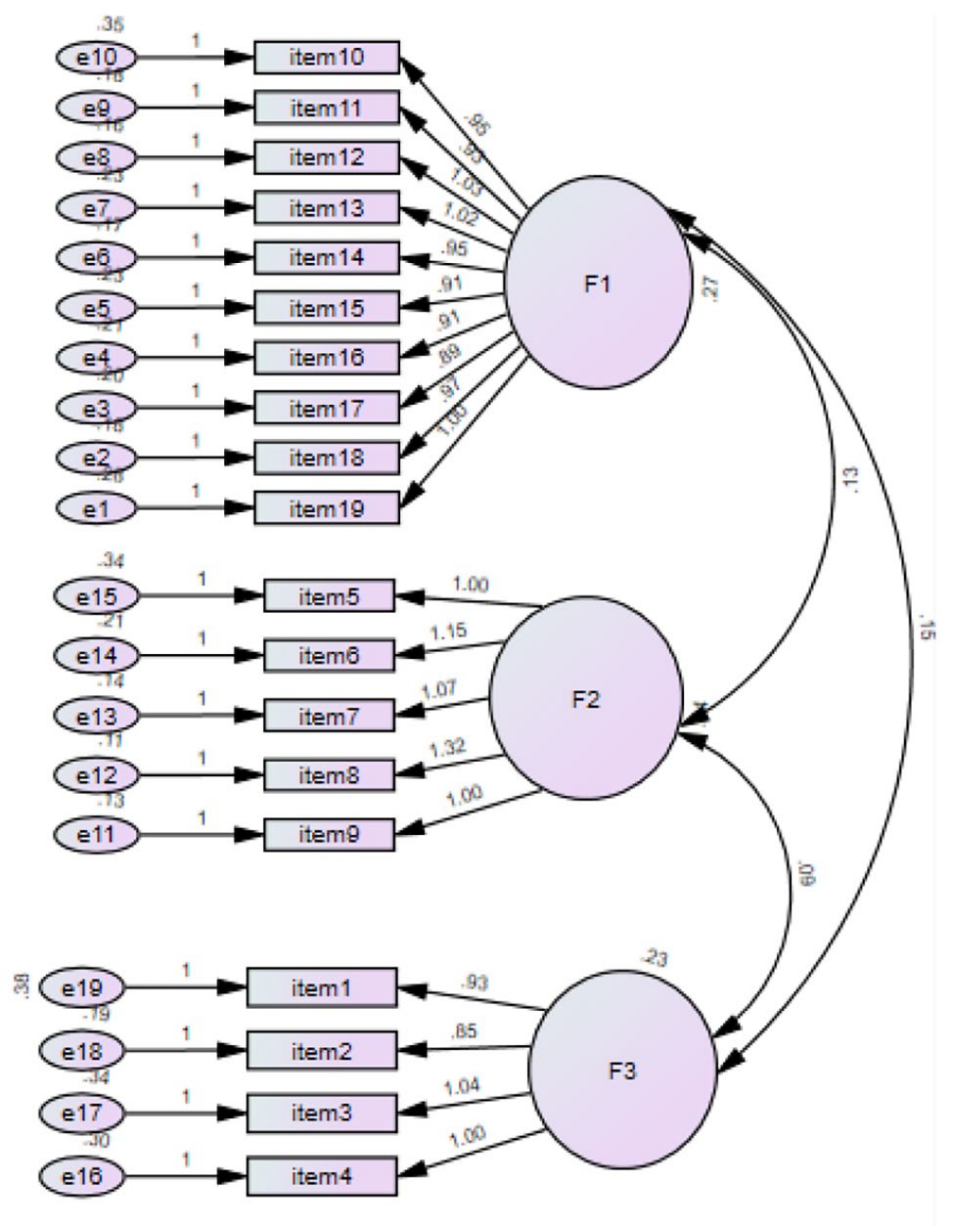
Path Diagram

CFA was conducted to test the three-factor structure obtained in the EFA. As a result of the CFA, the goodness-of-fit indices of the scale are as follows: χ2: 346.657; df:149; RMSEA = 0.069; IFI = 0.88; CFI = 0.92; IFI = 0.92 (Table 3). Factor loading of the scale items was above 0.30, and all the fit indices were satisfactory (Fig. 2).

**Table 3:** The Goodness of Fit Values of the Structural Model of the Educational Leadership Scale in Nursing Students

	Structural Model Values		Good Fit	Recommended Values
χ2/df	2.327		≤ 3	≤ 5
RMSEA	0.069		≥ 0.05	≤ 0.08
GFI	0.883		≥ 0.90	≥ 0.85
CFI	0.922		≥ 0.95	≥ 0.90
IFI	0.923		≥ 0.95	≥ 0.90
		**χ2: 346.657, df:149, p:0.00**		

After this stage, the items of each factor were examined and their sub-scale were labeled. In this context; The items in the first factor were labeled as “Visionary Leadership” sub-scale, the items in the second factor were labeled as “Instructional Leadership” sub-scale, and the items in the third factor were labeled as “Scientific Leadership” sub-scale (Table 4).

**Table 4:** Educational Leadership Scale For Nursing Students

Factor 1. Visionary Leadership (α = 0.92)	Factor Loading
Item 21 (10) I believe that the standards of National Core Program for Nursing should follow to plan educational program	0.650
Item 22 (11) I believe that standards of International nursing education program should follow to prepare the educational program.	0.777
Item 24 (12) I believe that nursing educational program should design in a way that facilitate learning.	0.778
Item 26 (13) I believe that technology should be used in vocational training.	0.741
Item 27 (14) I believe that the most appropriate assessment method should be used in the evaluation of vocational training.	0.733
Item 30 (15) I think it is very important to adopt professional ethics in nursing training.	0.672
Item 32 (16) I believe that learning module should be designed in accordance to nursing professional ethics.	0.729
Item 33 (17) I think it is important to develop and improve the communication skills of nurse candidates.	0.709
Item 35 (18) I think it is essential to develop cooperative skills in nurse candidates.	0.668
Item 36 (19) I think classroom education should be conducted in a democratic climate.	0.650
**Factor 2. Instructional Leadership (α = 0.82)**	
Item 13 (5) I believe that the field related learning modules should design in a way that develop interest for nurse candidates.	0.506
Item 17 (6) I believe that educational materials should be designed in accordance to learning objectives.	0.695
Item 18 (7) I believe that the most appropriate method should be chosen to determine the training needs of nurse candidates.	0.788
Item 19 (8) I believe that the learning progress of nurse candidates should be evaluated frequently.	0.807
Item 20 (9) I believe that the training contents should be designed in accordance to the needs of nursing profession.	0.759
**Factor 3. Scientific Leadership (α = 0.74)**	
Item 1 (1) I believe that I will be a professional role model for nursing profession candidates.	0.695
Item 2 (2) I frequently update my knowledge regarding my profession.	0.793
Item 3 (3) I follow the scientific activities related to my field.	0.707
Item 4 (4) I am willing to share my field related knowledge.	0.644

### Internal consistency reliability

Cronbach’s alpha analysis was used for confirming the reliability of the developed scale. Cronbach’s alpha is a measure of internal consistency. The Cronbach’s alpha of the total scale was good (α = 0.92). The Cronbach’s alpha for factor 1 was 0.92, for factors 2 was 0.82 and factor 3 was 0.74 (Table 4). The relation between the scale and its sub-scale was evaluated with the Pearson Product Moment correlation analysis technique, there was a statistically positive correlation between the total score of the scale and all sub-scales scores (p < 0.001).

### Stability Reliability

The stability reliability of the scale was evaluated by the test-retest method. Test-retest was carried out for reliability analysis of the scale concerning time, and the scale was administered to the 30 students from the population with an interval of 2 weeks. If a high coefficient of reliability was found with the Retest Method, it means that there is a stability between the scores obtained from the two administrations of the test. The mean value of the scale was found to be 86.76 ± 6.54 in the first application, and 85.73 ± 6.96 in the second application. Between two measurement relationship was analyzed using a paired-group t-test and Pearson correlation test. When the mean scores obtained from the test-retest were compared with the t-test (significance test of the difference between the mean of two dependent groups) independent groups. There was no significant correlation between the mean scores of the two measurements (r:0.894, p > 0.001).

### Scoring and evaluation of the scale

The scale is a 5-point Likert scale, and the total score that can be taken between “5” Strongly Agree and “1” Never Disagree statements is between 19 and 95 points. As the score of the scale increases, the educational leadership levels of nursing students increase positively. There are no inverse items in the scale.

## Discussion

The scale was developed as a self-report tool that evaluates the educational leadership tendencies of nurse candidates. In this respect, its most basic contribution in the academic sense is to bring it into the literature as a valid and reliable new measurement tool. The scale also helps to identify the relevant development areas as it enables the determination of the current educational leadership tendencies of the nurse candidates. In this sense, it can be thought that the scale is an important needs analysis tool in terms of curriculum development activities. The Educational Leadership Scale for Nursing Students consisted of 19 items and three factor structure. The three sub-scales created were named after the remaining 19 items were divided into subscales. While the first subscale created with items is called “Visionary Leadership”, the second subscale created is called “Instructional Leadership”, the third subscale created is called “Scientific Leadership”. This scale was developed in the perspective of Bloom’s Taxonomy. Taxonomy is a strategy that will facilitate and systematize learning. For this reason, this systematic strategy was used on the theoretical ground in the development of a self-report tool that will help develop educational leadership competency in nursing. The leadership characteristics that the educational leadership scale wanted to evaluate were divided into three sub-scales with statistics, these factors were named as “scientific, instructional and visionary” leadership with the consensus of the field experts (1 Curriculum Development Specialist, 5 Nurses), and then cognitive, affective and behavioral according to the fields in Bloom’s Taxonomy were classified as. Accordingly, while the “scientific and visionary” subscales of leadership included items from the “cognitive, affective and behavioral” domain outcomes, “affective and behavioral” domain outcomes were included in the “instructional leadership” subscale.

The construct validity of the scale was evaluated with the results of exploratory factor analysis and confirmatory factor analysis. Considering the fit indices obtained as a result of the exploratory factor analysis and confirmatory factor analysis results, it was seen that the fit indices gave good results as a result of the three sub-scale models.

In the first stage of the research, literature was reviewed for educational leadership scales. As a result of reviewing, there was no valid and reliable scale to evaluate the educational leadership for nursing students. This scale is believed to be so important for identifying, developing and maintaining the educational leadership of nursing students.

Two main features sought for a scale to be good are validity and reliability. Validity is about whether the items in a scale measure the same feature within the same structure. Reliability, on the other hand, is the consistency between independent measurements of individuals [37, 41].

Content and construct validity were used for scale validity. Content validity was used for the suitability of all items in the scale. With content validity, scale items were sent to the experts, and opinions were evaluated. Davis technique was preferred in the evaluation. In this technique, it is considered good that the items have a value of 0.80 within the scope of the content validity index [39, 42] The CVI values of all items were 0.80 and above. This finding showed that the content validity of the scale was good.

For the construct validity of the scale, first EFA and then CFA was performed. As a result of the EFA, a three-factor structure was observed in the scale. The load of the items in the final version of the scale, which consists of three factors and 19 items, is between 0.80 and 0.50.

When the literature is examined, factor loading values of 0.45 and above is an accepted value for scale items. The 3 sub-scale structure explains 58.64% of the total variance. The higher the ratio of total variance explained in the scale, the stronger the factor structure. However, it is stated in the literature that this value can be between 40 and 60 [43].

CFA was conducted to test the accuracy of the structure revealed by EFA. The fit indices that should be reported in confirmatory factor analysis are very diverse, but there is no consensus on which of these fit indices are accepted [44]. In this study, Considering the fit indices of the scale; Since χ2 / df value is below 3, it is a good fit, a GFI value of 0.88 is still an acceptable fit, a CFI value of 0.92 is a good fit, an IFI value of 0.92 is a good fit, and a RMSEA value of 0.06. showed good compatibility. These values showed good agreement. As a result, these fit indices revealed that the model had a good fit.

To verify the reliability of the developed scale, Cronbach’s α was calculated. Cronbach’s alpha coefficient measures the internal consistency, or reliability, of a set of survey items. This method is used to help determine whether of items consistently measures the same characteristic. The Cronbach’s Alpha reliability coefficient was found to be 0.92. Although it is accepted that the Cronbach’s Alpha coefficient is between 0.60 and 0.80, this value approaching indicates that the reliability of the scale is high [45]. With the results obtained, it has been found that the scale is reliable and valid with its 3 sub-scale structures.

Stability in reliability is a measure of the repeatability of a test over time, that it gives the same results whenever it is used [43]. The stability of the scale is accepted if the correlation coefficient between test-retest scores is 0.70 and more and positive. In the scale, there was no statistically significant difference between test and retest mean scores in all main subscales (r:0.894, p > 0.001). Therefore, the scale had stability in all dimensions as there is no significant difference between the test-retest scores and there is a strong or very strong positive correlation.

### Limitations

This study, aimed at developing a scale of educational leadership for nursing students and examining its validity and reliability, has certain limitations. Firstly, the present scale was applied to nursing student studying at a University of one country. This approach to data collection doesn’t represent all nursing programs in the country. Secondly, For this study, only one sample was used and this is one of the future limitation of the study. So more studies need to be conducted in various nursing groups to confirm the psychometry of the newly developed instrument for generalizability. In future comparative study between different countries in different nursing groups could be conducted to know their perspectives regarding scientific, instructional and visionary leadership.

## Conclusion

The present study explained the steps we took to develop a new tool to measure the educational leadership levels of nursing students. The validation approaches we used (checking the content validity of the items, piloting the scale, and subscale using explanatory factor analysis) provided justification for the content validity of the newly developed scale.

## Data Availability

The data that support the fndings of this study are available from the cor‑responding author upon reasonable request.
